# Non-specific physiological background effects of acupuncture revealed by proteomic analysis in normal rats

**DOI:** 10.1186/1472-6882-14-375

**Published:** 2014-10-05

**Authors:** Yu-Dong Xu, Yu Wang, Gyoung-Hee Park, Lei-Miao Yin, Jun Ran, Yan-Yan Liu, Yong-Qing Yang

**Affiliations:** Laboratory of Molecular Biology, Shanghai Research Institute of Acupuncture and Meridian, Shanghai University of Traditional Chinese Medicine, Shanghai, China; Yue Yang Hospital, Shanghai University of Traditional Chinese Medicine, Shanghai, China

**Keywords:** Acupuncture, Non-specific effect, Proteomics

## Abstract

**Background:**

The total effects of adequate real acupuncture treatment consist of pathologic-specific and non-specific physiological effects. The latter may be the fundamental component of the therapeutic effects of acupuncture. This study investigated the physiological background effects of acupuncture in normal rats treated with acupuncture.

**Methods:**

Manual acupuncture was performed on normal rats at experienced acupoints, GV14 (*Dazhui*), BL12 (*Fengmen*) and BL13 (*Feishu*), once every other day for two weeks. The proteomic profile of rat lung tissue was examined using 2-DE/MS-based proteomic techniques. Gene Ontology (GO) enrichment and the Kyoto Encyclopedia of Genes and Genomes (KEGG) pathway were analyzed for differentially expressed proteins using the WebGestalt toolkit.

**Results:**

In total, 25 differentially expressed protein spots were detected in the 2-DE gels. Among these spots, 24 corresponded to 20 unique proteins that were successfully identified using mass spectrometry. Subsequent GO and KEGG pathway analyses demonstrated that these altered proteins were mainly involved in biological processes, such as ‘protein stabilization’, ‘glycolysis/gluconeogenesis’ and ‘response to stimulus’.

**Conclusions:**

Our study indicated the non-specific background effects of acupuncture at acupoints GV14, BL12 and BL13 likely maintained internal homeostasis via regulation of the local stimulus response, energy metabolism, and biomolecule function balance, which may be important contributors to the therapeutic effects of acupuncture.

## Background

Acupuncture is part of Traditional Chinese Medicine (TCM) that has been used in the treatment and prevention of disease for more than 2000 years. The theory of acupuncture holds that there are different types of “*Qi*” (pronounced “chee” and usually translated as energy) moving through the human body along a network of channels or pathways called meridians, and blocks and/or disruptions of *Qi* are thought to cause health problems. A well-trained acupuncturist attempts to correct imbalances of the *Qi* and recover the equilibrium between physical, emotional and spiritual aspects of the individual by inserting needles into specific cutaneous points of the body.

Clinical trials and experimental investigations support the use of acupuncture for disease treatment, and these studies have facilitated its acceptance into clinic practice in most countries. The World Health Organization endorses acupuncture for at least two dozen conditions [1], and the clinical disease spectrum of acupuncture in China includes 411 western medicine diseases and 50 TCM syndromes [2], which indicates the applicability of acupuncture for many different diseases. Acupuncture effects are biological phenomena that must devolve from physiological and/or psychological mechanisms with some biological foundations [3]. However, acupuncture is also a complex intervention in which specific and non-specific effects are inextricably intertwined [4]. The total effects of an adequate real acupuncture treatment consist of pathologic-specific and non-specific physiological effects that are likely due to physiological processes triggered by the overall therapeutic context, namely the physiological background effect. The non-specific physiological effect of acupuncture is an important contributor to the therapeutic effects for various diseases. It is necessary to delineate the various components of acupuncture effects to better understand the biological foundations of its therapeutic effects.

Many recent publications have explored the therapeutic effects of acupuncture, but surprisingly few insightful studies have dealt with the physiological background effects of acupuncture. Our previous study demonstrated that acupuncture had an immunomodulatory effect on inflammatory cells and cytokines that were associated with an improvement in general well-being [5, 6]. We preliminarily analyzed the gene expression profile of acupuncture in normal rats using serial analyses of gene expression (SAGE) [7]. Experienced and effective acupuncture points, GV14 (*Dazhui*), BL12 (*Fengmen*) and BL13 (*Feishu*), were selected in the present study for acupuncture in normal rats, and a 2DE/MS-based proteomic analysis of the lung proteome was performed from acupuncture-treated normal rats to further elucidate the non-specific physiological background effects of acupuncture.

## Methods

### Animals and acupuncture procedure

The Committee on the Ethics of Animal Experiments of Shanghai University of Traditional Chinese Medicine approved all animal studies and procedures (approval ID: 08001), and all studies were conducted in accordance with the regulations set forth by the State Science and Technology Commission. Pathogen-free, male Sprague-Dawley (SD) rats (four weeks old, 110–130 g, SLAC Laboratory Animal Co. Ltd., Shanghai, China) raised in a pathogen-free rodent facility and provided with food and water *ad libitum* were randomly divided into two groups: normal control rats (NC, *n* = 15), and normal rats treated with acupuncture (AC, *n* = 15). All rats were kept in animal facilities approved by the Shanghai Committee for the Accreditation of Laboratory Animals for at least one week before the experiments began.

**Figure 1:**
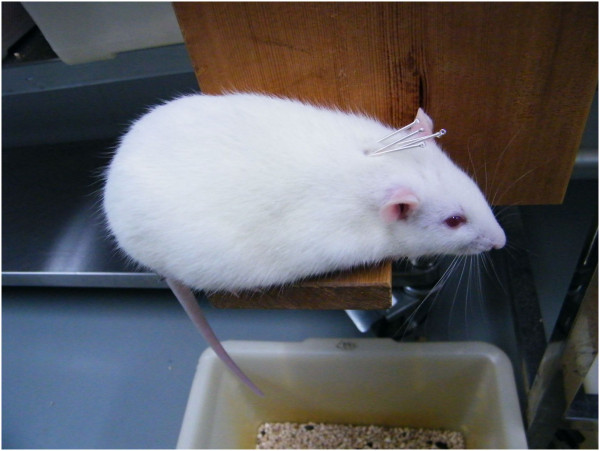
**Rats on the suspended shelf.** For the convenient manipulation of the acupuncture points on the back, rats were placed on the suspended shelf (50 × 45 mm, approximately 50 cm high from the ground), which calmed the rats and allowed them to stand still without anesthesia.

The acupuncture points GV14 (*Dazhui*, between the C7 and T1 vertebrae), bilateral BL12 (*Fengmen*, foveola laterally between the T2 and T3 vertebrae) and bilateral BL13 (*Feishu*, foveola laterally between the T3 and T4 vertebrae) were selected based on the theory of traditional Chinese medicine for the treatment of asthma [6]. Manual acupuncture was performed once every other day for two weeks. Disposable stainless needles (13 mm long, 0.30 mm in diameter, Suzhou Medical Appliance Factory, Suzhou, China) were inserted through the skin to a depth of approximately 5 mm. The needles were twisted approximately 360° evenly at the rate of 60 times/min for 20 s, manipulated every 5 min and withdrawn after 20 min. Rats were placed on a suspended shelf (50 × 45 mm, approximately 50 cm high from the ground, Figure 1) to minimize stress and conveniently manipulate the acupuncture points on the back, which calmed the rat and eliminated the need for anesthesia [8]. All rats were allowed to acclimate to the facility for one week, during which time they were not be experimentally manipulated. The same experienced practitioner performed all needle manipulations gently and firmly in a room without noises or sudden movements.

### Lung tissue sample preparation

The excised right lobe of the lung was rinsed free of blood in ice-cold saline and immediately frozen in liquid nitrogen. Protein from each lung tissue was extracted using a ReadyPrep Sequential Extraction Kit (Bio-Rad, Hercules, CA) with the following additions to Reagent 1 just before use: PMSF (1 mM), DNase (RNase-free; 20 μg/mL), and RNase (5 μg/mL). Protein concentrations were determined using a modified Bradford assay. For the 2-DE analysis, protein extracts from animals in the same group were pooled equally according to protein quantity and stored at -80°C until use.

### 2-DE analysis

Protein (100 μg) was loaded onto 17 cm IPG strips with a linear separation range of pH 3–10 (Bio-Rad, Hercules, CA), which were subsequently rehydrated for 12 h at 50 V, 20°C. First-dimensional isoelectric focusing (IEF) was performed in a Protean IEF Cell (Bio-Rad). The IEF voltage was raised using a rapid ramp to 10,000 V, and strips were run at 20°C with a current limit of 50 mA/strip until reaching 60,000 Vh. The focused IPG strips were equilibrated at room temperature in buffer (50 mM Tris–HCl pH 8.8, 6 M urea, 20% glycerol, and 2% SDS) with 2% DTT (w/v) for 15 min followed by a second equilibration in the same buffer containing 2.5% iodoacetamide (w/v) for 15 min. The strips were placed on a 13% T polyacrylamide gels and embedded in a 1% agarose stacking gel. The second-dimensional SDS-PAGE was performed at 24 mA per gel until the bromophenol blue dye front reached the bottom of the gel. Gels were run in triplicate for each sample and stained with silver nitrate solution. For image analysis, gels were scanned at high resolution using a GS-800 densitometer (Bio-Rad), and PDQuest software version 7.1 (Bio-Rad) was used to detect expression alterations in protein spots. Automatic spot detection and gel matching was followed by a manual validation of the matched and unmatched protein spots. The intensity volumes of individual spots were normalized by the total intensity volume of all spots present in each gel (%V). Only those protein spots with intensity alterations ≥ two-fold (*t* test, *p* < 0.05) were considered to demonstrate prominent differential expression.

### In-gel digestion and LC-MS/MS analysis

Protein spots with significant alterations in expression were manually excised from 2-DE gels, destained for 20 min with equal volumes of 30 mM potassium ferricyanide and 100 mM sodium thiosulfate at room temperature, and washed with Milli-Q water until the gels were destained. Spots were maintained in 0.2 M NH_4_HCO_3_ for 20 min and lyophilized. Each spot was digested overnight in 12.5 ng/mL trypsin in 0.1 M NH_4_HCO_3_. Peptides were extracted three times with 50% ACN, 0.1% TFA. Digested proteins were identified using a Finnigan LTQ mass spectrometer (ThermoQuest, San Jose, CA) coupled to a Surveyor HPLC system (ThermoQuest). First, a Microcore RP column (C18 0.15 mm × 120 mm; ThermoHypersil, San Jose, CA) separated the protein digests. Solvent A was 0.1% formic acid, and solvent B was 0.1% formic acid in 99.9% ACN. The gradient was held at 2% solvent B for 15 min and increased linearly to 98% solvent B over 90 min. Peptides were eluted from a C18 microcapillary column at a flow rate of 150 μL/min and electrosprayed directly into the LTQ mass spectrometer with an applied spray voltage of 3.2 kV. The full scan ranged from *m/z* 400 to 2000. Protein identification using MS/MS raw data was performed with SEQUEST software (Thermo Finnigan) by searching against the Swiss-Prot rat protein database. Identification results were filtered with Xcorr (1 + ≥ 1.9, 2 + ≥ 2.2, 3 + ≥ 3.75) and DelCn (≥0.1).

### Bioinformatic analysis

The Directed Acyclic Graph (DAG) of the enriched Gene Ontology (GO) biological process (BP) categories and Kyoto Encyclopedia of Genes and Genomes (KEGG) pathway analysis of differentially expressed proteins were performed using the bioinformatics tool WebGestalt (WEB-based GEne SeT AnaLysis Toolkit, http://bioinfo.vanderbilt.edu/webgestalt/) to highlight potential biological processes affected by acupuncture [9]. WebGestalt queries were performed using lists of Uniprot protein IDs. The entire rat genome was used as a reference set for the identification of enriched GO and KEGG pathway terms (i.e., GO terms with a number of associated genes significantly higher than expected). The hyper-geometric statistical method test was used, and only enriched GO sets (hypergeometric; *P* < 0.05) containing 2 or more genes were included in the analysis.

### Statistical analysis

The quantities of all detected protein spots in the two groups were compared using Student’s *t*-test in PDQuest 2-DE gel analysis software. Bioinformatic analysis was evaluated using a hypergeometric test against the entire rat genome in WebGestalt. The *p* value was adjusted for multiple comparisons, and a *p* value < 0.05 was considered an enriched GO category.

## Results

### Changes in lung proteome of normal rats treated with acupuncture

The proteins in samples from each group were measured using 2-DE at least three times, followed by verification that the same protein patterns were obtained. In general, we distinctly detected approximately 700–800 protein spots in each 2-DE gel and acquired a high overlapping rate (>85%) for spots in these parallel gels. Image analysis of the 2-DE gels revealed that acupuncture at specific points induced lung proteome changes in normal rats (NA group). In total, 25 protein spots showed consistently differential expression between the NC group and the NA group in all three parallel experiments. A total of 24 protein spots were successfully identified using LC-MS/MS, and these spots corresponded to 20 unique proteins. Among the 24 spots, 10 were down-regulated and 11 were up-regulated in the acupuncture normal rats (Table 1). Both up- and down-regulated expression patterns were observed for the actin alpha skeletal muscle protein, which may reflect differential post-translational modification of the same protein.

**Table 1:** **The 25 differentially expressed proteins between NC and NA rats**

Spot No.	Protein name	Gene symbol	Swiss-prot No.	Theoretical Mr (kDa)/PI	Pept./Cov% ^a)^	Fold change ^b)^
1	Alpha-1-antiproteinase	Serpina1	P17475	46.1/5.7	6/11.68%	↓2.62
2	Gama-enolase	Eno2	P07323	47.0/7.0	2/5.07%	↓3.2
3	Calreticulin	Calr	P18418	48.0/4.3	49/36.06%	↓5.78
4	Phosphatidylethanolamine-binding protein 1	Pebp1	P31044	20.8/5.5	9/25.13%	↓2.51
5	Actin, alpha skeletal muscle	Acta1	P68136	42.0/5.2	29/22.02%	↓2.49
6	Actin, cytoplasmic 1	Actb	P60711	41.7/5.3	13/10.67%	↑4.17
7	Beta-enolase	Eno3	P15429	47.0/7.0	2/5.07%	↓3.29
8	Creatine kinase B-type	Ckb	P07335	42.7/5.4	2/4.46%	↑3.18
9	Actin, alpha skeletal muscle	Acta1	P68136	42.0/5.2	8/11.94%	↑4.58
10	serum albumin precursor	Alb	P02770	68.7/6.0	7/6.58%	++ ^**c)**^
11	Heat shock cognate 71 kDa protein	Hspa8	P63018	70.9/5.4	35/30.03%	↑2.26
12	Transgelin 2	Tagln2	Q5XFX0	23.4/7.6	15/32.38%	++
13	Creatine kinase B-type	Ckb	P07335	42.7/5.4	2/4.46%	↑2.27
14	Fibrinogen beta chain	Fgb	P14480	54.2/7.9	55/45.3%	-- ^**d)**^
15	Heat shock cognate 71 kDa protein	Hspa8	P63018	70.9/5.4	19/18.89%	↑3.16
16	Glyceraldehyde-3-phosphate dehydrogenase	Gapdh	P04797	35.8/8.1	20/20.12%	↑3.36
17	22 kDa protein	—	—	21.9/9.8	7/25.00%	↑2.21
18	Heterogeneous nuclear ribonucleoproteins A2/B1	Hnrnpa2b1	A7VJC2	37.4/9.0	46/36.83%	↑3.1
19	Tubulin alpha-1A chain	Tuba1a	P68370	50.1/4.9	2/5.32%	↓17.16
20	Phosphoglycerate mutase 1	Pgam1	P25113	28.8/6.7	11/16.93%	--
21	Serum albumin precursor	Alb	P02770	68.7/6.0	39/30.59%	↑2.5
22	NG,NG-dimethylarginine dimethylaminohydrolase 2	Ddah2	Q6MG60	29.7/5.7	6/11.93%	++
23	Annexin A5	Anxa5	P14668	35.7/4.9	14/23.20%	++
24	Advanced glycosylation end product-specific receptor	Ager	Q63495	42.7/5.8	7/8.21%	↑2.37
25	Peroxiredoxin-6	Prdx6	O35244	24.8/5.6	13/26.34%	--

^a)^The number of matching peptides (Pept.) and percentage of the total amino acid sequence covered by the peptides (% Seq. Cov) from the mass mapping experiments.

^b)^The change level in optical density of the protein spots in NA rats compared with NC rats.

^c)^++ represents spots detected in gels from the NA samples but not detected in NC samples, which was considered up-regulated in the NA group.

^d)^-- represents spot detected in gels from the NC samples but not detected in NA samples, which was considered down-regulated in the NA group.

### Bioinformatic analysis of proteins differentially expressed following acupuncture

**Figure 2:**
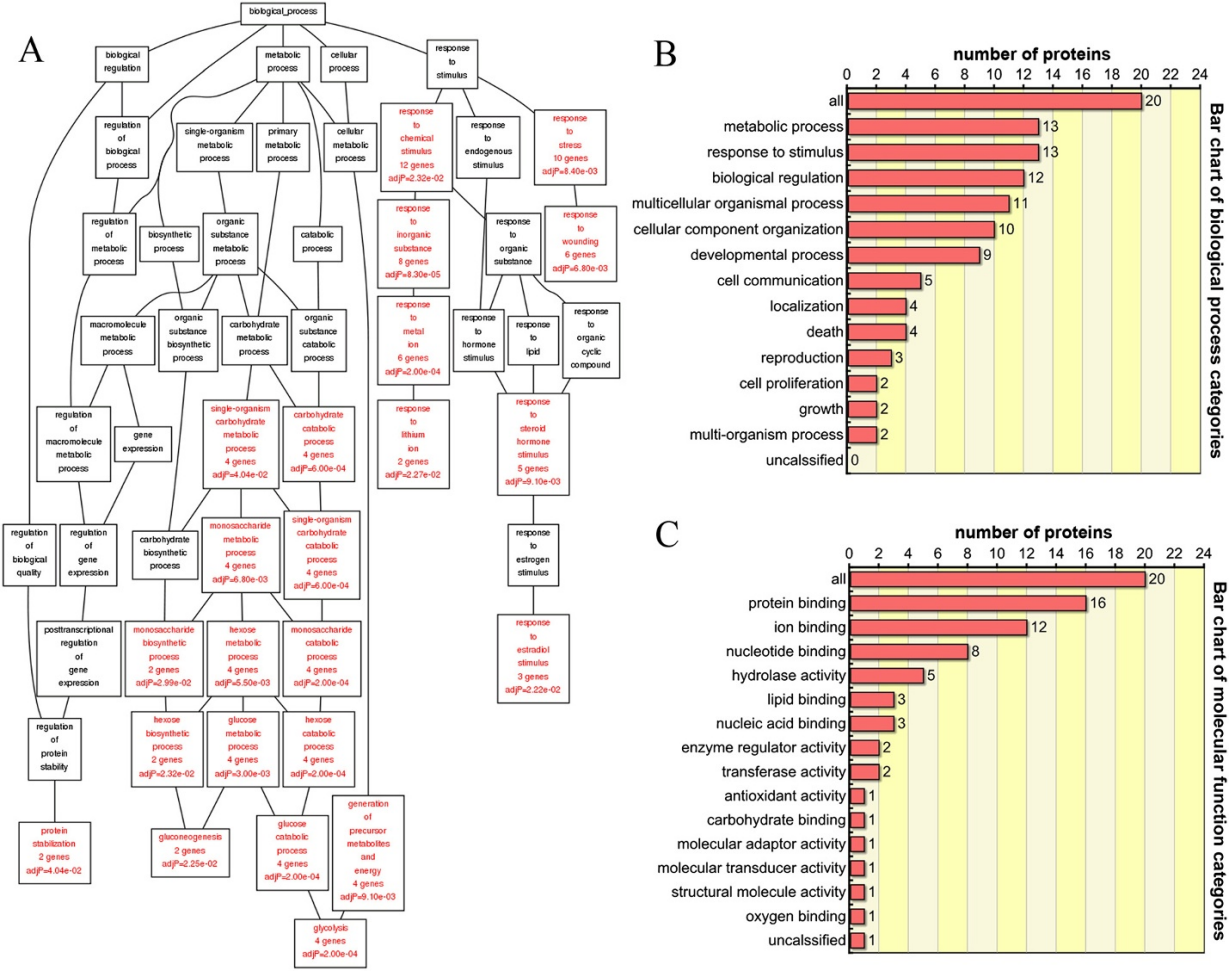
**The identified proteins were analyzed according to Gene Ontology (GO) enrichment using WebGestalt (WEB-based GEne SeT AnaLysis Toolkit). (A)** Directed Acyclic Graph (DAG) of the enriched GO categories under Biological Process. Each node shows the name of the GO category, the number of gene/proteins in the category and the *P* value indicating the significance of enrichment. **(B)** and **(C)** GO classification from the Biological Process and Molecular Function ontology enrichment. The number of proteins enriched in each GO term is shown at the right side of the bars.

Differentially expressed proteins after acupuncture were classified according to biological process or molecular function using the WebGestalt software package on the basis of hypergeometric tests as described in the Methods section. Resulting BP networks are shown as Directed Acyclic Graphs (DAG), which are color-coded for *P* values < 0.05 (Figure 2A). Particular enrichment was found for stability regulation and stimulation-associated proteins and proteins involved in energy metabolism regulation. Significantly enriched GO categories under Biological Process and Molecular Function are indicated in Figure 2B and C. In the biological process category, GO terms related to metabolic process (13 proteins), response to stimulus (13 proteins), biological regulation (12 proteins) and multicellular organismal process (11 proteins) were enriched. In the molecular function category, GO terms related to protein binding (16 proteins), ion binding (12 proteins), nucleotide binding (8 proteins) and hydrolase activity (5 proteins) were over-represented in lung proteome profiles. Pathway analysis using WebGestalt implicated several pathways in acupuncture treatment of normal rats, including the KEGG categories ‘Glycolysis/Gluconeogenesis’ and ‘metabolic pathways’ (Table 2). These results suggest the main effect of acupuncture on normal rats was the maintenance of internal homeostasis via regulation of local responses to stimulus, energy metabolism, and metabolic balance.

**Table 2:** **List of enriched KEGG pathways of the differentially expressed proteins**

KEGG pathway	Hits number	Corresponding gene symbol	Statistics for the enrichment of the pathway
Glycolysis/Gluconeogenesis	4	Eno3, Eno2, Gapdh, Pgam1	C = 78; R = 115.29; adjP = 2.78e-07
Metabolic pathways	6	Eno3, Eno2, Gapdh, Pgam1, Prdx6, Ckb	C = 1169; R = 11.54; adjP = 3.02e-05
Phagosome	3	Tuba1a, Actb, Calr	C = 185; R = 36.46; adjP = 0.0002
RNA degradation	2	Eno3, Eno2	C = 77; R = 58.40; adjP = 0.0007
Complement and coagulation cascades	2	Fgb, Serpina1	C = 69; R = 65.17; adjP = 0.0007
Protein processing in endoplasmic reticulum	2	Hspa8, Calr	C = 164; R = 27.42; adjP = 0.0024

## Discussion

Acupuncture is a complex intervention that always involves a spectrum of treatment factors (pathologic-specific effects) and associated effects (placebo, non-specific, context effects), which makes it extremely difficult to fully unravel the inner mystery of this biological phenomenon [10]. Numerous clinical studies have demonstrated pathologic-specific effects of acupuncture for the treatment of various diseases.

Acupuncture regulates autonomic nerve system functions (e.g., modulates blood pressure, heart rate, and sphincter intensity) [11, 12], immune system and inflammatory pathways [13, 14], and endocrine system functions, such as the release of endorphins [15] and NO [16], under different pathological conditions. However, few acupuncture studies focus on the physiological processes of this practice on healthy bodies, in which the functions of organs, tissues and cells are relatively balanced. The normal healthy body is an ideal state to study the physiological background effects of acupuncture, which are concealed by several active pathological processes during a disease state.

Traditional Chinese Medicine theory states that the effects of acupuncture include a rebalancing or restoration of normal function or flow of *Qi*, which is the vital energy or life force that provides function or motive force and flows within the body through a systemic network of meridians or pathways. *Qi* can become blocked, disrupted, or deficient due to imbalances or pathogenic influences, which lead to dysfunction and disease. Acupuncture modulates the flow of *Qi* through the meridians so that the main organs (*Zang-Fus*) re-establish homeostasis, as governed by the laws of *Yin-Yang* and the Five Elements [17]. The combined use of the experienced acupoints *Dazhui*, *Fengmen* and *Feishu* regulate *Qi* in the lung meridian, remove obstructions from the meridian, and disperse lung *Qi* to stop asthma [5]. However, the self-regulation effects of acupuncture from these acupoints on the healthy body, which is an ongoing maintenance of balance and harmony in the circulation of *Qi*, are not known.

Our previous study preliminarily analyzed the gene expression profile of acupuncture on normal rats using serial analyses of gene expression to explain the physiological background effects of acupuncture treatments. The results showed that the background effects of acupuncture included the regulation of biosynthesis, transportation and metabolism [7]. Our present study further characterized the proteomic profile of lung tissue in acupuncture-treated rats using two-dimensional electrophoresis and mass spectrometry. GO analysis suggested that proteins involved in metabolic process, stimulus response and biological regulation were regulated after acupuncture intervention. These regulated proteins included proteins with roles in ‘protein stabilization’, ‘glycolysis/gluconeogenesis’, and ‘response to stimulus’.

Protein stabilization includes any process that is involved in the maintenance of protein structure and integrity and the prevention of protein degradation or aggregation. In many cases, ion binding and protein binding are primary approaches for the maintenance of the structural and functional stability of protein molecules. GO analysis revealed that acupuncture regulated 16 protein-binding proteins and 12 ion-binding proteins. Glycolysis is a fundamental system for energy metabolism in most organisms, and it plays an essential role in energy homeostasis and the support of cell survival [18]. The identification of differentially expressed glycolytic proteins in the lung tissue proteome demonstrated the involvement of energy metabolism and energy balance within the background effects of acupuncture intervention. Another class of proteins that are regulated by acupuncture are involved in the responses to stimuli or stress. Stress is a well-known factor that may be defined as the response to any physical or psychological stimulus that threatens ongoing homeostasis [19]. The insertion of needles into the body of a rat and the placement of the rats on a suspended shelf were sufficient to cause physical or acrophobia-induced stress in this study. The expression of stress-related proteins, such as calreticulin and heat shock 70 kDa protein, may be another non-specific effect of acupuncture treatment.

## Conclusions

In summary, our study examined the lung proteome of normal rats treated with acupuncture using a 2-DE/MS based proteomic approach to delineate the non-specific effects of acupuncture using biological experimental evidence. Our results indicated that the background effect of acupuncture at GV14, BL12 and BL13 modulated biological regulatory processes, including protein stability, stress responses and metabolic systems, such as energy metabolism and responses to steroid hormone stimuli, which could maintain or promote physiological homeostasis in the body. Our study demonstrated a non-specific physiological background effect in addition to the specific effects of needle stimulation, which may be an important contributor to the therapeutic effects of acupuncture.
